# Author Correction: Biosynthesis of cannabinoid precursor olivetolic acid in genetically engineered *Yarrowia lipolytica*

**DOI:** 10.1038/s42003-022-04377-7

**Published:** 2022-12-22

**Authors:** Jingbo Ma, Yang Gu, Peng Xu

**Affiliations:** 1 Department of Chemical, Biochemical and Environmental Engineering, University of Maryland, Baltimore County, Baltimore, MD 21250 USA; 2 Present Address: College of Biological and Pharmaceutical Engineering, West Anhui University, Lu’an, Anhui 237012 China; 3 Present Address: School of Food Science and Pharmaceutical Engineering, Nanjing Normal University, Nanjing, 210023 China; 4 Present Address: Department of Chemical Engineering, Guangdong Provincial Key Laboratory of Materials and Technologies for Energy Conversion (MATEC), Guangdong Technion-Israel Institute of Technology, Shantou, Guangdong 515063 China

**Keywords:** Metabolic engineering, Biosynthesis

Correction to: *Communications Biology* 10.1038/s42003-022-04202-1, published online 12 November 2022.

The original version of this Article had several incorrect references in the following paragraphs of the Results and Discussion section:

Last sentence of subheading “**Biosynthesis of olivetolic acid in engineered** ***Y. lipolytica***”:

“The resulting strain YL101 only produced 0.11 mg/L OLA after 96 h cultivation (Fig. 2b), which is comparable to the initial OLA production in *S. cerevisiae*^19^.”

The *correct* version of this paragraph should appear as:

“The resulting strain YL101 only produced 0.11 mg/L OLA after 96 h cultivation (Fig. 2b), which is comparable to the initial OLA production in *S. cerevisiae*^**17**^.”

Second paragraph of subheading “***Screening enzymes to improve pool size of hexanoyl-CoA***”:

“ [..] We also removed the C-terminal peroxisome targeting sequence 1 (PTS1) and overexpressed the codon-optimized CsAAE3 in *Y. lipolytica*^20,21,22^. […] The LvaE gene encoding the short-chain acyl-CoA synthetase of *Pseudomonas putida* KT2440 (PpLvaE, PP_2795 with uniport ID Q88J54), which is reported to active with C4-C6 carboxylic acids, was also selected^19^. […]. The overexpression of CsAAE1 in *S. cerevisiae* only showed a twofold increase in OLA titer^19^. […]”

The *correct* version of this paragraph should appear as:

“ [..] We also removed the C-terminal peroxisome targeting sequence 1 (PTS1) and overexpressed the codon-optimized CsAAE3 in *Y. lipolytica*^**19-21**^. […] The LvaE gene encoding the short-chain acyl-CoA synthetase of *Pseudomonas putida* KT2440 (PpLvaE, PP_2795 with uniport ID Q88J54), which is reported to active with C4-C6 carboxylic acids, was also selected^**22**^. […]. The overexpression of CsAAE1 in *S. cerevisiae* only showed a twofold increase in OLA titer^**17**^. […]”

These have now been corrected in the PDF and HTML versions of the Article.

