# Supplementary material for: Biosynthesis of cannabinoid precursor olivetolic acid in genetically engineered Yarrowia lipolytica
Source: Commun Biol. 2022 Nov 12;5:1239. doi: 10.1038/s42003-022-04202-1 (PMC9653464; doi:10.1038/s42003-022-04202-1)
Supplement: Supplementary file 2 — Supplementary Information [file 42003_2022_4202_MOESM2_ESM.pdf]

Supplementary Information for

Biosynthesis of cannabinoid precursor olivetolic acid in genetically engineered *Yarrowia lipolytica*

Jingbo Ma[1,2], Yang Gu[1,3] and Peng Xu[1,4*]

[1]Department of Chemical, Biochemical and Environmental Engineering, University of Maryland, Baltimore County, Baltimore, MD 21250, USA

[2]Present address: College of Biological and Pharmaceutical Engineering, West Anhui University, Lu'an, Anhui 237012, China

[3]Present address: School of Food Science and Pharmaceutical Engineering, Nanjing Normal University, Nanjing 210023, China

[4]Present address: Department of Chemical Engineering, Guangdong Provincial Key Laboratory of Materials and Technologies for Energy Conversion (MATEC)，Guangdong Technion-Israel Institute of Technology, Shantou, Guangdong 515063, China

[*] Corresponding authors Tel: + 86-0754-88077163. E-mail: peng.xu@gtiit.edu.cn (PX).

Supplementary Table 1. Primers used in this study

Primers	Sequence
CsOAC-F	ccgaccagcactttttgcagtactaaccgcaggccgtgaaacaccttattgtcctc
CsOAC-R	ggacaggccatggaactagtcggtaccttatttacgaggggtgtagtcgaaaataag
CsOLS-F	ccgaccagcactttttgcagtactaaccgcagaatcacctccgggcagaagg
CsOLS-R	ggacaggccatggaactagtcggtaccttaatacttaatggggacgcttc
CsOAC-Gly-F	ggaggaggtggtggtggtggtggaggtggcatggccgtgaaacaccttattgtcctc
CsOLS-Gly-R	acggccatgccacctccaccaccaccaccacctcctccatacttaatggggacgcttct
EcFadD-F	ccgaccagcactttttgcagtactaaccgcagaagaaggtttggcttaaccgttatcc
EcFadD-R	ggacaggccatggaactagtcggtacctcaggctttattgtccactttgccgcgcg
EcFadK-F	tccgaccagcactttttgcagtactaaccgcagcatcccacaggcccgcatctcgg
EcFadK-R	ggacaggccatggaactagtcggtaccttattcaatctcttcacagacatc
ScFAA2-F	tccgaccagcactttttgcagtactaaccgcaggccgctccagattatgcacttaccg
ScFAA2-R	ggacaggccatggaactagtcggtaccctaaagcttttctgtcttgactag
ylACC1-F	tccgaccagcactttttgcagtactaaccgcagcgactgcaattgaggacactaacacg
ylACC1-R	ggacaggccatggaactagtcggtacctcacaaccccttgagcagctcagcccg
ylACC1(S667A)-F	agttagacctcttgctgacggtggtattc
ylACC1(S667A)-R	aataccaccgtcagcaagaggtctaactc
ylACC1(S1178A)-F	gtctcgagctgatgccgtctccgacttttc
ylACC1(S1178A)-R	aagtcggagacggcatcagctcgagacac
ylFAA1-F	ccgaccagcactttttgcagtactaaccgcaggtcggatacacaatttcctcaaagcc
ylFAA1-R	ggacaggccatggaactagtcggtaccctaagactgctcgtagcactcatcaatttc
pTEF-Rvs	ccggatggccagacaaagaaaca
XPR2-Fw	gtaaatagaaaatctggcttgtaggtggcaaaat
Ku70_DwF	gtccggagcggccgcGCATGCaagtcgacaCTAGGGAGGCACATCTAAACGAATAACG
Ku70_DwR	gttacatccttttatcagacatacctaggAGTGAACGACCAAGACTAAAGGGTG
ku70_UpF	ccctaaatttgatgaaagcctaggCGACTTGATGTTTAGAGTGTCCAGATCC
ku70_UpR	taatgtatgctatacgaagttatTTTCAAAAAGCGGCGGTTCGTG
ku70_ChkF	GCCAAGTTCTCTTTCCCCTACATG
ku70_ChkR	CTTCAGTAACTGGGCCCACGC
YliC05907-UpF	tggcatccctaaatttgatgaaagcctaggACAGTTCTCTTCTCCTTGTTGAGATATAC
YliC05907-UpR	acttcgtataatgtatgctatacgaagttatGGTGGAGTCATGTGAATTGAGCGCGGTG
YliC05907-DwF	cgtccggagcggccgcGCATGCaagtcgacAGAGTATAGTAATGTATTATTTGCTTAGG
YliC05907-DwR	cctatgttacatccttttatcagacatacctaggCCGAACCAAGGAGATGATCAAGTCC
YliC05907-ChkF	ATTGCCGAGATTTCCGCAAAAACCTGAAG
YliC05907-ChkR	AAGAACAAGACCGACAACCTGCCTGTTGG
YliDGA1_DwF	gctagcgagacaataacggaggaGGAAAACTGCCTGGGTTAGGCAAAT
YliDGA1_DwR	atcagacatagcggccgcTCTCTGATGGCCTGGAGCGAG
YliDGA1_UpF	aatttgatgaaaggcggccgcATGCTGCGGGCGGATCCTGG
YliDGA1_UpR	tgtatgctatacgaagttatAGCTTTTGTTTTGTGTGACTTGTCTGT
YliDGA1_ChkF	GTTTATGCATTCTGTTGGACCTTAGTCTG
YliDGA1_ChkR	GTTATCTACCACGATTTTTTGGTTTCTGAGGC

YliDGA2_DwF	gctagcgagacaataacggaggaCATAACACTCATCAGTAGCCTTTACAGTGAT
YliDGA2_DwR	ccttttatcagacatagcggccgcTTGCTCTTGTAATTCCATAGATAATATATACGAAA
YliDGA2_UpF	aaatttgatgaaaggcggccgcCTTGGGAGTGTATTTGGAAAATGACTTGG
YliDGA2_UpR	tgtatgctatacgaagttatTTTGCGGGCGGTACGGGTACA
YliDGA2_ChkF	CTATCGCCCCAAAGTGTTTCTAGCA
YliDGA2_ChkR	GAGATGGCATGCCAACGTTGAC
ylPEX10-F	tccgaccagcactttttgcagtactaaccgcagtggggaagttcacatgcattcgctgg
ylPEX10-R	ggacaggccatggaactagtcggtaccttatctgataggcaacaagttctgctctc
EcLpdA-F	ccgaccagcactttttgcagtactaaccgcagagtactgaaatcaaaactcaggtc
EcLpdA-R	GGACAGGCCATGGAACTAGTCGGTACCTTACTTCTTCTTCGCTTTCGGGT
EcAceF-F	ccgaccagcactttttgcagtactaaccgcaggctatcgaaatcaaagtaccggac
EcAceF-R	GGACAGGCCATGGAACTAGTCGGTACCTTACATCACCAGACGGCGAATGTC
EcAceE-F	ccgaccagcactttttgcagtactaaccgcagtcagaacgtttcccaaatgacgtg
EcAceE-R	ggacaggccatggaactagtcggtaccttacgccagacgcgggttaactt
EcLplA-F	ccgaccagcactttttgcagtactaaccgcagtccacattacgcctgctcatctc
EcLplA-R	GGACAGGCCATGGAACTAGTCGGTACCCTACCTTACAGCCCCCGCCATCCATG
ylMAE1-F	ccgaccagcactttttgcagtactaaccgcagttacgactacgaaccatgcgaccc
ylMAE1-R	GGACAGGCCATGGAACTAGTCGGTACCCTAGTCGTAATCCCGCACATGGATG
ylANT1-F	tccgaccagcactttttgcagtactaaccgcaggcagctatttccaaagactatgttc
ylANT1-R	ggacaggccatggaactagtcggtaccttatcccttgatcaaggtgggggccttcatg

Supplementary Table 2. Plasmids used in this study

Plasmids	Characteristics
pYLXP'	pYaliA1 vector backbone with leucine marker and Ampicillin resistance gene
pYLXP'-CsOLS	pYLXP' containing gene CsOLS
pYLXP'-CsOAC	pYLXP' containing gene CsOAC
pYLXP'-CsOLS-CsOAC	pYLXP' containing gene CsOLS and CsOAC
pYLXP'-CsOLS-Gly-CsOAC	pYLXP' containing gene CsOLS and CsOAC with Glycine linker
pYLXP'-CsOAC-Gly-CsOLS	pYLXP' containing gene CsOAC and CsOLS with Glycine linker
pYLXP'-CsAAE1	pYLXP' containing gene CsAAE1
pYLXP'-CsAAE3	pYLXP' containing gene CsAAE3
pYLXP'-EcFadD	pYLXP' containing gene EcFadD
pYLXP'-EcFadK	pYLXP' containing gene EcFadK
pYLXP'-ScFAA2	pYLXP' containing gene ScFAA2
pYLXP'-ylFAA1	pYLXP' containing gene ylFAA1
pYLXP'-PpLvaE	pYLXP' containing gene PpLvaE
pYLXP'-CsOLS-CsOAC-CsAAE1	pYLXP' containing gene CsOLS, CsOAC and CsAAE1
pYLXP'-CsOLS-CsOAC-CsAAE3	pYLXP' containing gene CsOLS, CsOAC and CsAAE3
pYLXP'-CsOLS-CsOAC-EcFadD	pYLXP' containing gene CsOLS, CsOAC and EcFadD
pYLXP'-CsOLS-CsOAC-EcFadK	pYLXP' containing gene CsOLS, CsOAC and EcFadK
pYLXP'-CsOLS-CsOAC-ScFAA2	pYLXP' containing gene CsOLS, CsOAC and ScFAA2
pYLXP'-CsOLS-CsOAC-ylFAA1	pYLXP' containing gene CsOLS, CsOAC and ylFAA1
pYLXP'-CsOLS-CsOAC-PpLvaE	pYLXP' containing gene CsOLS, CsOAC and PpLvaE
pYLXP'-ylACC1	pYLXP' containing gene ylACC1
pYLXP'-ylACC1^{S667A}	pYLXP' containing gene ylACC1^{S667A}
pYLXP'-ylACC1^{S667A,S1178A}	pYLXP' containing gene ylACC1^{S667A,S1178A}
pYLXP'-CsOLS-CsOAC-PpLvaE-ylACC1	pYLXP' containing gene CsOLS, CsOAC, PpLvaE and ylACC1
pYLXP'-CsOLS-CsOAC-PpLvaE-ylACC1^{S667A,S1178A}	pYLXP' containing gene CsOLS, CsOAC, PpLvaE and ylACC1^{S667A,S1178A}
pYLXP'-ylPEX10	pYLXP' containing gene ylPEX10
pYLXP'-SeACS^{L641P}	pYLXP' containing gene SeACS^{L641P}
pYLXP'-EcPDH-EcLplA	pYLXP' containing gene EcPDH and EcLplA
pYLXP'-ylPEX10-SeACS^{L641P}-EcPDH-EcLplA	pYLXP' containing gene ylPEX10, SeACS^{L641P}, EcPDH and EcLplA
pYLXP'-ylMAE1-ylANT1-McMAE2	pYLXP' containing gene ylMAE1, ylANT1 and McMAE2
pYLXP'-ylPEX10-SeACS^{L641P}-EcPDH-EcLplA-ylMAE1-ylANT1-McMAE2	pYLXP' containing gene ylPEX10, SeACS^{L641P}, EcPDH, EcLplA, ylMAE1, ylANT1 and McMAE2
pYLXP'-loxP-ura	pYLXP'containing the loxP-URA-loxP cassette
pYLXP'-loxP-hygr	pYLXP'containing the loxP-hygr-loxP cassette
pYLXP'-Cre	pYLXP'containing gene Cre
pURLA	*Ku70* site integration plasmid
pURLB	*YALI0C05907g* site integration plasmid

pYLXP'-loxP-ura-ylDGA2	pYLXP'-loxP-ura containing gene ylDGA2 deletion cassette
pYLXP'-loxP-hygr-ylDGA1	pYLXP'-loxP-hygr containing gene ylDGA1 deletion cassette
pURLA-CsOLS-CsOAC-PpLvaE-ylACC1	pURLA containing gene CsOLS, CsOAC, PpLvaE and ylACC1
pURLB-CsOLS-CsOAC-PpLvaE-ylACC1	pURLB containing gene CsOLS, CsOAC, PpLvaE and ylACC1

NOTE: Cs, *Cannabis sativa*; Ec, *Escherichia coli*; Sc, *Saccharomyces cerevisiae*; yl, *Yarrowia lipolytica*; Pp, *Pseudomonas putida* KT2440; Se, *Salmonella enterica*; Mc, *Mucor circinelloides*.

Supplementary Table 3. Strains used in this study

Strains	Characteristics
po1g	Wild-type strain W29 (ATCC20460) derivate; W29 ΔmatAΔxpr2-332 Δaxp-2 Δleu2-270 pBR platform
po1f	po1g derivate; Further deletion of gene ura; po1g Δura3
po1fk	po1f derivate; Further deletion of gene ku70; po1f Δku70::loxP
YL100	po1fk with the empty plasmid pYLXP’
YL101	po1fk with plasmid pYLXP'-CsOLS-CsOAC
YL102	po1fk with plasmid pYLXP'-CsOLS-Gly-CsOAC
YL103	po1fk with plasmid pYLXP'-CsOAC-Gly-CsOLS
YL104	po1fk with plasmid pYLXP'-CsOLS-CsOAC-CsAAE1
YL105	po1fk with plasmid pYLXP'-CsOLS-CsOAC-CsAAE3
YL106	po1fk with plasmid pYLXP'-CsOLS-CsOAC-EcFadD
YL107	po1fk with plasmid pYLXP'-CsOLS-CsOAC-EcFadK
YL108	po1fk with plasmid pYLXP'-CsOLS-CsOAC-ScFAA2
YL109	po1fk with plasmid pYLXP'-CsOLS-CsOAC-ylFAA1
YL110	po1fk with plasmid pYLXP'-CsOLS-CsOAC-PpLvaE
YL111	po1fk derivate; Further deletion of genes ylDGA2; po1fk ΔylDGA2::loxP
YL112	YL111 derivate; Further deletion of genes ylDGA1; po1fk ΔylDGA2ΔylDGA1::loxP
YL113	YL111 with plasmid pYLXP'-CsOLS-CsOAC-PpLvaE
YL114	YL112 with plasmid pYLXP'-CsOLS-CsOAC-PpLvaE
YL115	po1fk with plasmid pYLXP'-CsOLS-CsOAC-PpLvaE-ylACC1
YL116	po1fk with plasmid pYLXP'-CsOLS-CsOAC-PpLvaE-ylACC1^{S667A,S1178A}
YL117	po1fk derivate; Further integration of genes CsOLS-CsOAC-PpLvaE-ylACC1 at *YALI0C05907g* site; po1fk CsOLSCsOACPpLvaEylACC1::loxP
YL118	po1fk derivate; Further integration of genes CsOLS-CsOAC-PpLvaE-ylACC1 at *ku70* site; po1fk CsOLSCsOACPpLvaEylACC1::loxP
YL119	po1fk derivate; Further integration of genes CsOLS-CsOAC-PpLvaE-ylACC1 at pBR platform; po1fk CsOLSCsOACPpLvaEylACC1::Leu
YL120	YL117 with the empty plasmid pYLXP’
YL121	YL118 with the empty plasmid pYLXP’
YL122	YL118 with plasmid pYLXP'-ylPEX10
YL123	YL118 with plasmid pYLXP'-SeACS^{L641P}
YL124	YL118 with plasmid pYLXP'-EcPDH-EcLplA
YL125	YL118 with plasmid pYLXP'-ylPEX10-SeACS^{L641P}-EcPDH-EcLplA
YL126	YL118 with plasmid pYLXP'-ylMAE1-ylANT1-McMAE2
YL127	YL118 with plasmid pYLXP'-ylPEX10-SeACS^{L641P}-EcPDH-EcLplA-ylMAE1-ylANT1-McMAE2
YL128	YL118 derivate; Further integration of genes ylPEX10-SeACS^{L641P}-EcPDH-EcLplA-ylMAE1-ylANT1-McMAE2 at pBR platform; YL118 ylPEX10SeACS^{L641P}EcPDHEcLplAylMAE1ylANT1McMAE2::Leu

Supplementary Note 1: Codon-Optimized gene sequences

>CsOLS

atgaatcacctccgggcagaaggccccgcatccgtcctggctatcggtactgcaaatcccgaaaacatccttctccaagacgagttccccgattat
tatttccgagtcaccaagtctgagcacatgactcaactcaaggagaaattccggaagatttgtgacaaaagcatgatccgtaagcggaactgtttcc
tgaatgaagagcacctgaagcagaatcctcgacttgttgaacacgagatgcagacccttgatgcacgtcaggacatgcttgttgtggaggttccca
agctgggtaaagatgcctgtgccaaggctatcaaggagtggggccagcctaaatcgaagatcacgcatctgattttcacctctgcctcgacaactg
atatgcccggagctgattatcactgtgcaaaactgctcggtctgagcccctctgtcaaacgggttatgatgtaccagctgggttgctacggcggtgg
aacggtgctgcgtatcgcaaaggatattgccgaaaataacaaaggcgccagagtccttgctgtgtgttgtgacattatggcctgtctcttccgggga
ccctccgaatccgatcttgaactgcttgtcggacaagcaatttttggtgatggcgcagccgccgttatcgttggtgcagagcctgatgaatctgtgg
gtgaaagacccattttcgagctcgtctcgacaggtcaaactatcctgcccaattcggagggcacaattggtggacatattcgtgaagcaggactga
ttttcgacctgcacaaagatgttcctatgcttatctccaataacatcgagaaatgccttatcgaggcctttacccctatcggcatttcggattggaattcg
atttttggatcacccatcccggaggaaaagcaatcctcgataaagtggaagaaaaacttcacctcaagtccgataagttcgtggactcccggcatg
tgctctctgaacatggaaatatgtcgtcctctactgtccttttcgtgatggacgaacttcggaagcggtccctcgaggaaggcaaaagcacaacag
gtgacggatttgaatggggtgttctctttggctttggccctggccttacggttgaacgagtcgttgttagaagcgtccccattaagtattaa

>CsOAC

atggccgtgaaacaccttattgtcctcaaattcaaggacgaaatcactgaggcacagaaggaggaatttttaaaacctacgtcaatctcgtcaatat
cattcctgctatgaaggatgtctattggggcaaagatgtgacccaaaaaaacaaggaagaaggatacacccacattgtcgaggttacattcgaga
gcgttgaaacgattcaagactacatcattcaccccgctcacgttggctttggtgacgtttaccgatccttttgggaaaagctccttattttcgactacac
ccctcgtaaa

>CsAAE1

atgggaaagaactacaagtcgcttgattccgtggtcgcctcggattttatcgctctgggtattacatcggaagtcgctgagactcttcatggccggct
cgcagaaattgtttgtaactatggagctgccacgcctcagacttggatcaacattgctaatcacattctctcccccgaccttcctttctccctgcatcaa
atgctcttctatggctgctacaaggatttcggtcctgcacctcctgcttggattcctgatcctgagaaagttaaatctactaacctcggtgcccttctgg
agaaacgtggtaaagaattcctgggtgtgaaatacaaggaccctatttcgtccttttctcacttccaagagttctccgttcgaaatcctgaggtctattg
gcgtacggtccttatggatgagatgaagatttctttttccaaagatcctgagtgcattctccgtcgagacgacattaataaccccggtggttccgaatg
gcttcctggtggatatctcaattccgctaaaaattgcctcaatgttaactccaataaaaagctgaatgatacgatgatcgtttggcgagacgaaggca
atgacgacctgccccttaacaaactcactctcgaccagctgcgaaaacgagtctggcttgtgggctacgcccttgaagagatgggtctcgaaaaa
ggatgtgccatcgctattgacatgcccatgcatgttgacgccgtggtgatctacctcgcaattgttcttgctggatacgtggtggtgagcatcgccga
cagcttttcggcacccgagattagcacgcggctcagactctcgaaagccaaagctatcttcacccaagatcatattatccgaggcaaaaaacggat
ccctctctattcgcgtgtggtcgaagcaaaaagccccatggccatcgttattccctgttctggctccaacatcggcgcagagcttagagacggcgat
atctcctgggactacttcctcgagagagctaaggagtttaagaactgtgaattcactgcacgagagcagcccgtcgatgcttacaccaacatcctgt
ttagctctggcaccactggtgaacctaaggcaatcccttggacccaagctacacctcttaaggctgcagcagatggttggtcgcatctcgacatcc
gaaagggcgatgttatcgtgtggcctactaaccttggctggatgatgggtccttggctcgtctatgcctctctgctcaacggtgccagcatcgccctt
tataacggtagccccccttgtctccggcttcgcaaagtttgtgcaagatgccaaggttacaatgcttggtgtcgtcccttctattgttagaagctggaaa
agcacaaattgcgtttccggctacgactggtctactattcggtgctttagcagctcgggtgaagcctcgaatgtggatgaatacctctggctgatgg
gtagagcaaattacaaacctgtcattgagatgtgtggcggaaccgagatcggaggagccttctcggctggatcctttcttcaagctcaatcccttag
ctctttttcttcgcagtgtatgggttgtacgctgtatattcttgataaaaatggataccccatgcctaaaaacaaacctggtattggagagcttgcccttg
gccctgtcatgttcggcgcttcgaaaacacttctgaacggcaaccaccacgacgtttatttcaagggtatgcccacgctcaacggtgaagtccttcg
acgtcatggcgatattttcgaactgacaagcaacggatactatcatgctcatggtagagccgatgacacgatgaacattggtggaatcaagatctct
tccatcgagattgaacgggtctgtaacgaagttgacgaccgtgtcttcgaaacgacggccattggagtgcctcctcttggtggtggtcctgagcag

ctcgttattttttcgttctcaaggattctaatgatacaactatcgacctgaaccagctgcggctgtctttcaatcttggactgcaaaaaaagctcaatcc
ccttttcaaagtcacgcgtgtggtgcctctctcgtcgctgcctcggactgccaccaataaaattatgcggcgggtgctccgacagcagttttcccattt
cgagtaa

>CsAAE3
atggagaagagcggttatggacgggatggaatctatcgttctctgcgtcccccccttcatctgcctaataacaataacctgagcatggtgtcgttcct
cttccgaaactcctcttcctatcctcagaaacccgcacttatcgactctgagacgaatcaaattctttccttttcccacttcaagtccacagtcatcaaag
tctcgcacggatttcttaaccttggaattaagaagaacgatgtcgttctgatttatgcccctaattcgatccacttccccgtttgctttctcggtattattgc
atcgggcgctattgccaccacgtccaatcctctctatactgtcagcgaactttctaagcaggtgaaggactcgaatcctaaactcattatcacggtgc
cccaactcctcgaaaaggtgaaaggattcaatctccctaccattctcattggccctgattcggaacaggaatccagcagcgataaggtcatgacatt
taatgacctcgttaaccttggtggctcttccggctctgaatttcccattgtggatgatttcaaacaatctgacacggccgcactcctttactcgagcgga
acaaccggaatgtcgaagggcgtcgttctcacgcacaagaatttcatcgcaagctctcttatggttaccatggagcaagacctggtcggtgaaatg
gacaacgtttttctttgttttcttcccatgttccacgttttcggtctcgctattattacttacgcccaacttcagcgtggaaataccgtcatttctatggccag
atttgatcttgaaaagatgctcaaggatgttgaaaagtacaaggtgactcacctctgggtcgttcctcccgtgatccttgccctttctaagaatagcat
ggttaaaaagtttaacctttctagcattaaatatatcggtagcggcgctgcacctcttggcaaggaccttatggaggaatgcagcaaagtcgtgccct
atggaatcgtggctcaaggctatggcatgaccgagacttgcggtattgtctctatggaagatattcgaggtggaaaacggaattctggtagcgcag
gcatgctcgcttcgggagttgaggctcaaattgtttccgttgataccctgaagcctctccctcctaaccagctcggagaaatttgggtgaaaggacc
caacatgatgcaaggatacttcaacaaccctcaggcaactaagctgacaattgacaagaaaggttgggtgcataccggagatctgggctactttga
cgaggacggacatctctacgtcgttgatcgaatcaaggaactcatcaaatacaagggcttccaagtcgcacccgctgagctcgaaggactgctgg
tcagccaccctgagattctggacgctgtcgtcattcccttccccgatgcagaagccggagaggttcctgttgcctacgtggtcagatcgcccaactc
gtccctgactgaaaatgatgtcaagaaatttatcgctggccaagtcgcttcttttaagcggctgcgaaaggtcactttcattaacagcgttcctaaaag
cgcttctggaaaaattctttaa

>PpLvaE
atgatggtgcccacgcttgagcatgagctggctcctaacgaggctaaccacgtccctctcagcccccttcgttcctgaagcgagcagcacaagt
gtatcctcaacgggatgctgtcatctacggtgcaagacgatactcgtatcgtcagcttcatgaacgttctcgagctcttgcttcggctcttgagcgggt
gggcgtccagcctggagagcgtgtcgctatcctggcccccaacattcctgaaatgctggaagcccattatggcgtgcccggagccggtgcagtg
ctcgtctgtattaacatccgtcttgagggccggtctattgccttcatcctccggcactgtgcagcaaaagtcctcatttgcgaccgtgagtttggtgct
gttgcaaatcaggcactcgctatgctggacgcacctcccctgctggtcggaatcgacgacgaccaagctgaacgggccgatctggcacacgatc
tcgactatgaagccttccttgcacagggagatcctgcacgtcctctgtctgcccccaaaacgagtggcagtctatcgccattaactatacttccgg
aacaacaggtgatcccaaaggagtcgttctgcaccaccgtggagcatatcttaacgcctgtgctggtgccctcatcttccaactgggcccccggtc
tgtgtatctgtggactcttcccatgttccattgcaacggatggtctcatacttgggctgtgactctctcgggcggtacgcacgtttgtcttcgaaaggtg
caacctgacgcaatcaatgccgccattgctgaacacgcagttacccatctgtcggccgcacccgtcgtcatgtccatgctcatccacgccgagca
cgcatcggcacctcccgttcctgtgtccgtgattacgggaggagctgcccccccttcggccgtcattgctgcaatggaagcacggggctttaatatt
actcacgcttacggaatgacagagtcgtatggaccttcgactctctgcctgtggcagcctggtgtcgacgagctgcccctggaagccagagccca
gtttatgtcgagacagggtgtcgctcacccccctcctcgaagaagccaccgtgctcgacacggatacgggacgacccgtccctgcagacggcctg
actctcggtgaactcgttgttcgtggaaacacagtcatgaaaggatacctccataatcccgaagcaactcgtgcagccctcgctaacggttggctg
cacacgggcgatctcgccgtgctccatctcgatggatatgttgaaattaaggatagagcaaaagatattatcatctcgggaggtgagaatatttcctc
gctcgagatcgaagaggttctgtaccagcaccccgaggtggtcgaggctgcagtcgttgccagacctgactctcgttggggcgagacgcccca
cgcattcgtcaccctcagagccgacgcactggcttccggtgacgatctcgtgagatggtgtagagaacgactggcccattttaaagctcctcggca
cgtgtctctggtcgatcttcctaaaacagccaccggaaaaatccaaaaatttgtcctccgtgaatgggcacgacagcaagaggcacagatcgcag
atgctgagcattaa

>SeACS^{L641P}
atgagccaaactcacaagcatgcaatccccgccaacattgccgaccgttgcctcattaaccctgaacagtacgaaactaagtacaaacaatccatc
aacgatcctgataccttttggggagagcagggtaaaattctggattggattacaccttatcagaaagtgaaaaatacatcttttgcccctggcaatgtc
tccatcaaatggtatgaagacggcaccctgaatctggctgctaattgtcttgaccgacacctgcaagaaaacggagatcggactgctattatttggg
aaggagatgacacgagccagtccaagcatatctcgtatcgggagctgcatcgagacgtgtgtcggtttgccaatactctcctcgaccttggaatta
aaaaaggtgatgtggtggccatctatatgcctatggttcctgaggccgctgtggctatgctggcctgtgcccgtattggtgctgtgcatagcgtcattt
ttggaggcttctcgcctgaggcagtcgcaggacggatcattgattcctcctcccggctggtgatcactgcagacgaaggagtccgggctggacg
atctattcccctgaagaagaatgtggacgacgcactcaagaaccctaacgtgacctctgttgagcatgtgatcgtgctgaaacgaacaggctctga
tattgattggcaagagggacgtgatctgtggtggcgtgacctgattgaaaaagcatcgcccgagcaccaacctgaggcaatgaatgcagaagac
cccctgttcatcctgtacacctcgggatctacaggtaaacccaaaggcgtgctccataccacgggtggataccttgtctacgccgcaactacgttca
agtacgtgtttgactatcatcccggagacatttactggtgtacagctgatgttggatgggttacaggtcattcctatcttctgtacggccctctcgcttgt
ggcgcaactacacttatgtttgagggtgttcctaattggcccacacctgctcgaatgtgccaggtggtggataaacaccaagtgaacattctgtaca
cagcccccacagccatccgggccctcatggccgaaggcgacaaagctatcgagggtacggaccgatcttcgctccggatccttggctctgttgg
agagcctatcaatcctgaagcttgggagtggtattggaagaagattggcaaggagaagtgccccgtggttgacacatggtggcagacggagac
gggaggattcatgatcacccctctccccggagccatcgaactgaaagccggaagcgcaactcgtcccttttttggcgttcaacccgcccttgtgga
caacgagggacacccccaggagggcgctacagaaggtaatcttgttatcacagactcctggcccggacaggctcgaacactctttggcgatcac
gaacggtttgagcaaacctacttcagcacatttaaaaacatgtacttttccggagatggtgcacggcgggacgaagacggctactactggattacg
ggaagagttgatgatgttctcaacgtctctggccaccgacttggaacggcagaaatcgaatctgccctcgtcgcccatcctaagattgccgaagcc
gcagttgtcggaattccccacgccatcaaaggtcaagccatctacgcttacgtgactctcaaccatggtgaagaacctagccccgagctgtacgc
cgaggtcagaaactgggttcgaaaggaaattggtcctcttgcaacgcctgatgtccttcattggactgattccctgcctaaaacccggagcggaaa
gattatgcgacgaatccttcggaagatcgccgctggtgacacgtcgaacctcggcgatacctctacactggccgaccccggagtggtcgaaaaa
cctcttgaggagaaacaagcaatcgcaatgccctcctaa

>McMAE2
atgtcgcccatcatcgatttcgtgcgacgtcagcttagctctacaaaacttcacgaggaacagcaaaccgccactacgaacgatctcgtgtcgcgtt
ccggatatcttaacgagggcaaatatgaagttcggcttaactgcatcaacgctggttgtcttcaaaaaaaactcaactatatcggtactgcaatggac
cctgctaaacgtcaaagacttggcctgaatggtcttctccccgccggtgtcgaaacccttgaaattcagaaggcccgagccctgcgtgtcctgcgtt
ccaaacacaatctcctcgagaaatatattcttatggcccaactgcgtacaacgaacgttcggcttttctataagattgttattgacgaactcgaaacgg
tgcaacttgcccccgtcatttacactcccaccgtcggaaccgcttgtctggagtatagcactatctacccctttctcgcagcacctggtgttcccgatg
gactttacctcacaaaggcagagctccccgaactgtgccagacaatccgtaactacagacccactgacactgaaggttttgagcccgagattgcc
gttatcagcgacggttctcggatcctgggtcttggcgacctgggcaccaacggtatgggtattcctatgggtaaacttcaactgtacgtggccggc
gctggaatcgaccctcgacgtacactccccatcattctcgaccttggtaccaataatgaaaaactgctcaacgatgagttttatatcggccttcggca
gaaaagacctaatgacgaagagttttaccagacagttgataccgtgcttactgccctccatactgtgtatcccaacctcctcattcagttcgaggact
ggtcgtcggaacacgcattcggccttctggagaaataccagaatcaaatgctttgcttcaacgacgatatccaaggtacaggagccgtgatcctgt
ccggtgtcatcaacgcaattcggaaagtcgaaaaggagaatcaggtttcgcccagagaccaccgtattgtcttttacggagcaggctctgccgcc
atcggcgttgctcgtcagatccagtcctatttccagatcgaacacaacatgacggaggaagaggcaaagcacgtgttctggatcgtcgattctaaa
ggcctcgtcaccacaacacgtggtgataagctggctcagcataaagtctactacgctagaggcgataacgagggacaacagtataaagagctga
tcgacattgttaactataacctttatagcctcattggactcagcagcacgactggtgcatttaatacgcaggttctggaacgacttgcttcccttaatga
gcagcccattgtttttcctctgagcaatcctgcaacgcaggctgagtgtacctttgaacaggctatggaggctaccaataataaggttatctttgccag
cggaacagctttccccgcttatacgattaaatctacaggagaggtgaatacgcccggtcaaggcaataacatgtacatttttcccggccttggtctcg
gcgcatgccttgctaatcctgcacacttcgaccgtatgatctacgaagcctccaaagcactcgcagattccctgacagaagaagaaattagcaaag

cttggctctatccttctctcaactatcggagcgtttccgctatcgtcgccgccgcagtttgccaagagactctgaacgaaaatcttgccacctcccaa
gctatgatgacacaatgtaaatcgcatgaggatattcttgactatgttagcgctcacatgtggagccctgattacggaaataataattccaaccagca
agccggtaagtaa

Supplementary Note 2: LC-MS characterization of OLA standards and samples

Olivetolic Acid by LC-MS/MS

For: Jingbo Ma (CBEE)
January 7, 2020

Report prepared by: M. LaCourse

Date: January 7, 2020

Report reviewed by: Joshua Wilhide

Date:

Objective & Solution Preparation

- The goal of this analysis is to utilize LC-MS/MS to determine the olivetolic acid concentration in 3 samples
- Sample and standard preparation:
  - Samples were analyzed as provided by transferring to autosampler vials
    - Sample 3 was non-homogenous and thus vortexed thoroughly before analysis but still separated quickly
  - A standard curve was created in methanol from the provided 10 ppm stock following the scheme below:

C1 (ppb)	V1 (uL)	C2 (ppb)	V2 (uL)	Diluent (uL)
10,000	150	1000	1500	1350
10,000	50	500	1000	950
1,000	250	250	1000	750
1,000	100	100	1000	900
1,000	50	50	1000	950
1,000	10	10	1000	990

Instrumentation & Method

Instrumentation:
- Qsight LX50:
  - PerkinElmer UHPLC Precision Sampling Module
  - Perkin Elmer UHPLC Solvent Delivery Module
  - PerkinElmer UHPLC Column Temperature Module
- Perkin Elmer QSight 210 Mass Spectrometer
- Column: Agilent Zorbax Eclipse XDB-C18 2.1 x 50 mm, 1.8 µm (P/N 981757-902)

HPLC Method: Olivetolic Acid V1
- Injection Volume: 10.0 µL
- Column Temperature: 40°C
- Run time: 5.00 min
- Flow: 0.3 mL/min
- Isocratic: (40:60 water:methanol)

MS Method: Olivetolic Acid V1
- Drying Gas: 80.0
- HSID Temperature (°C): 320.0
- Nebulizer Gas 1: 100.0
- ElectroSpray V1 Pos: -3000.0
- Source 1 Temperature (°C): 200.0

Olivetolic Acid (negative mode)
- Q1 Mass: 223.5
- Q2 Mass: 180
- Dwell Time: 795 ms
- Resolution (Q1:Q2): Unit_Unit
- CE: 22, EV:-30, CCL2: 20

Calibration Curve

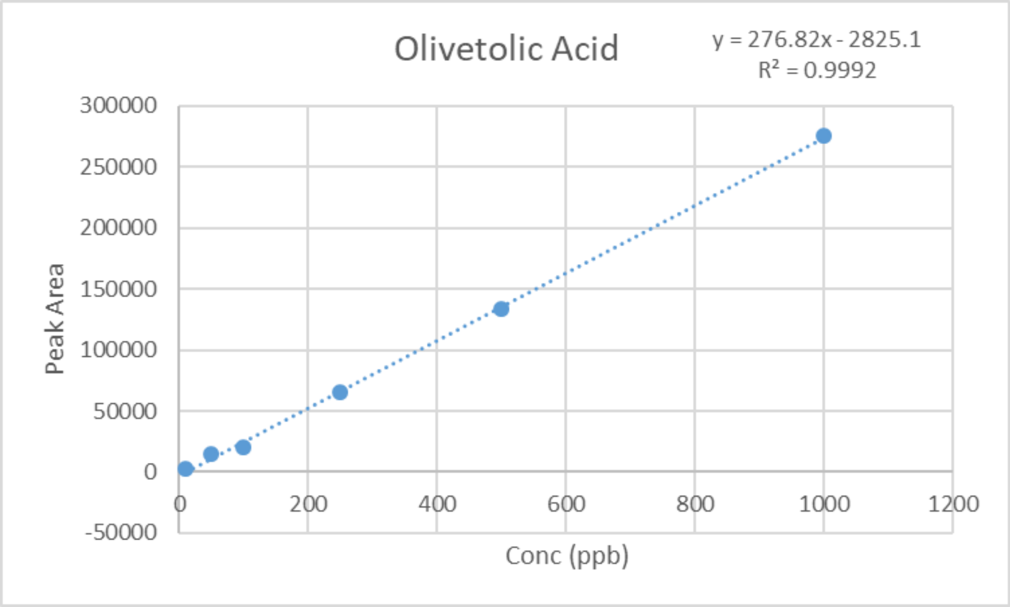

Methanol Blank

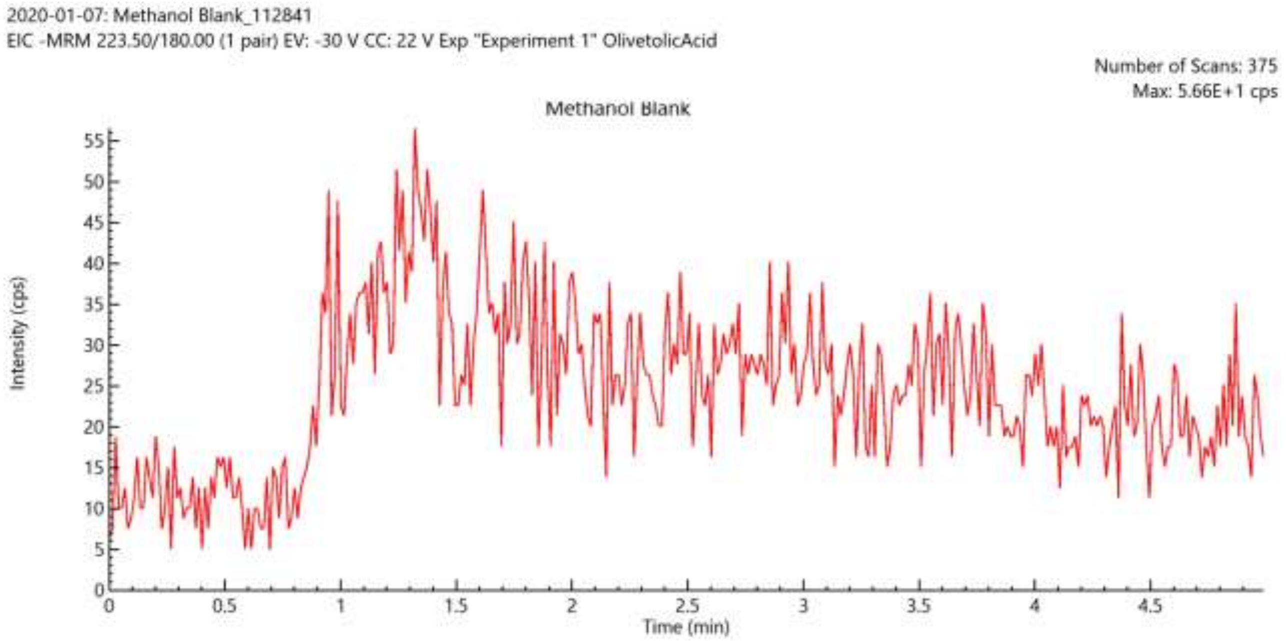

500 ppb Olivetolic Acid Standard

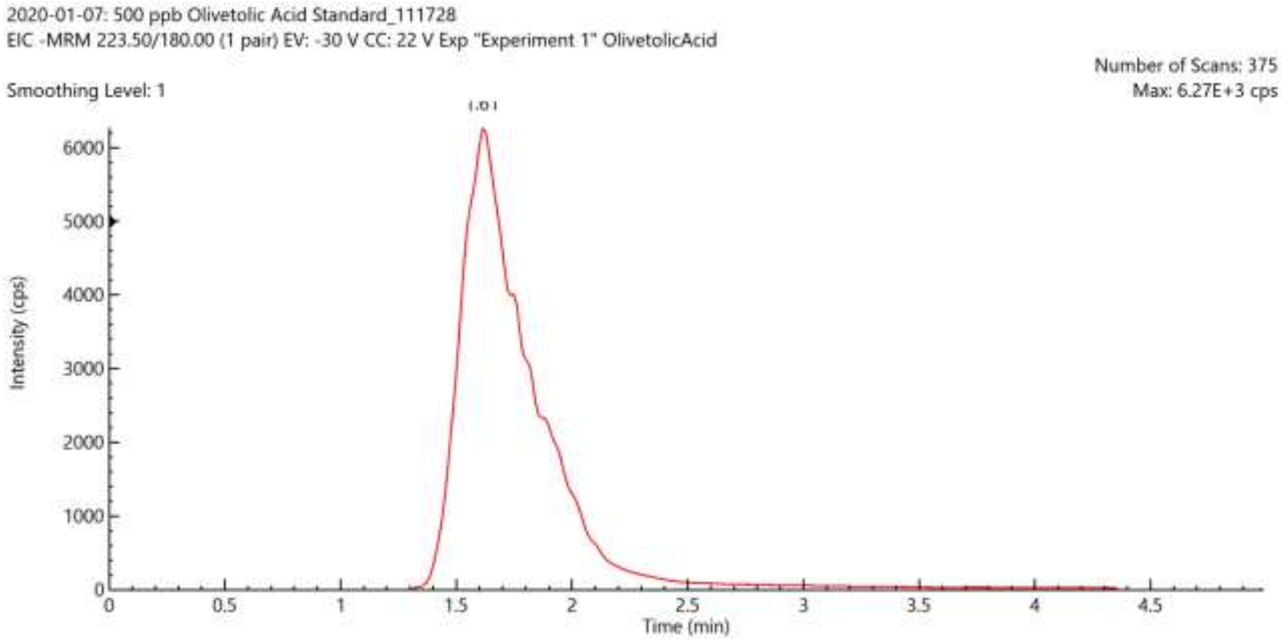

Sample 1

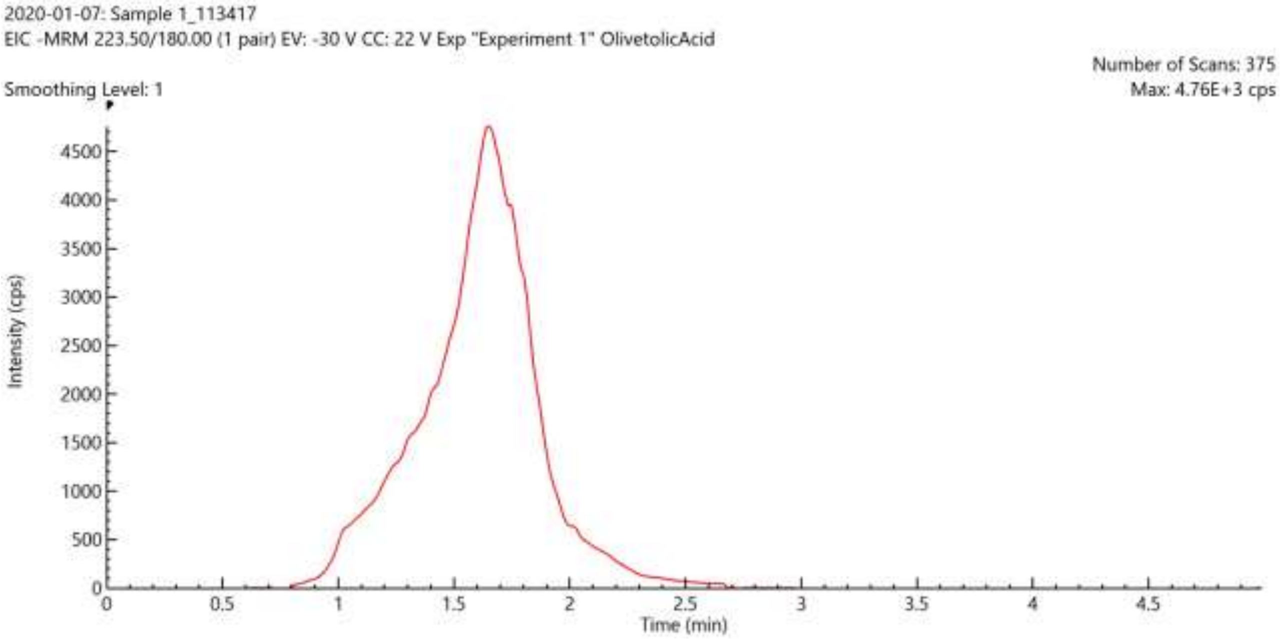

Sample 2

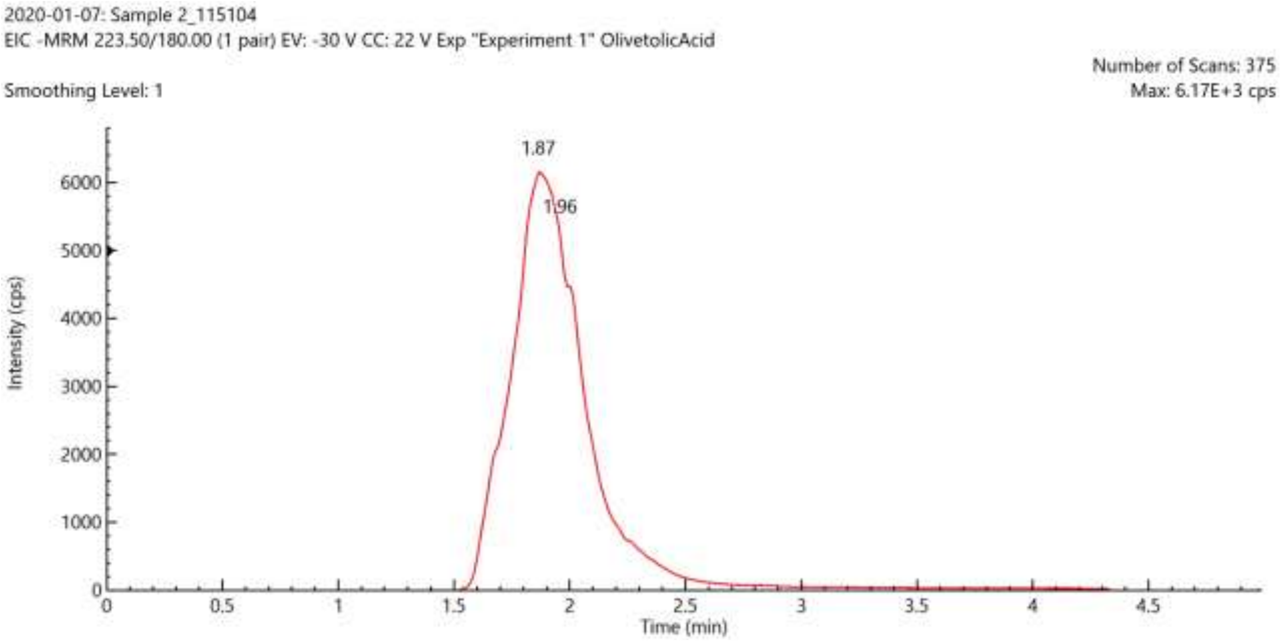

Sample 3

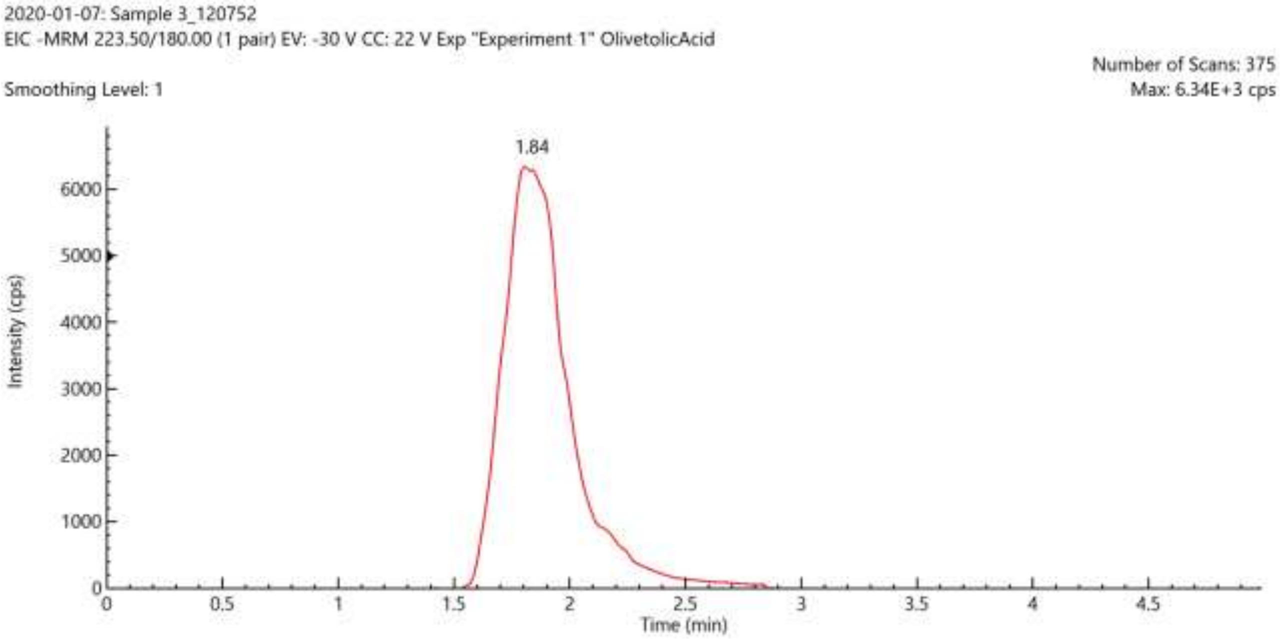

Results

- See corresponding spreadsheet for all raw values and calculations

- Determined concentrations:
  - Sample 1: 649 ppb
  - Sample 2: 533 ppb
  - Sample 3: 467 ppb

Olivetolic Acid by MS/MS

MCAC

For: Jingbo Ma (CBEE)
January 14, 2020

Report prepared by: M. LaCourse

Date: January 14, 2020

Report reviewed by: Joshua Wilhide

Date:

Instrumentation & Method

Instrumentation:
- Perkin Elmer QSight 210 Mass Spectrometer

MS Method:
- Drying Gas: 80.0
- HSID Temperature (^{o}C): 320.0
- Nebulizer Gas 1: 100.0
- ElectroSpray V1 Pos: -3000.0
- Source 1 Temperature (^{o}C): 0.0

Olivetolic Acid (negative mode)
- CE: 22
- Fragmentation:
    - EV:- 30
    - CCL2: 20

Mass Spectrum 1 ppm Olivetolic Acid

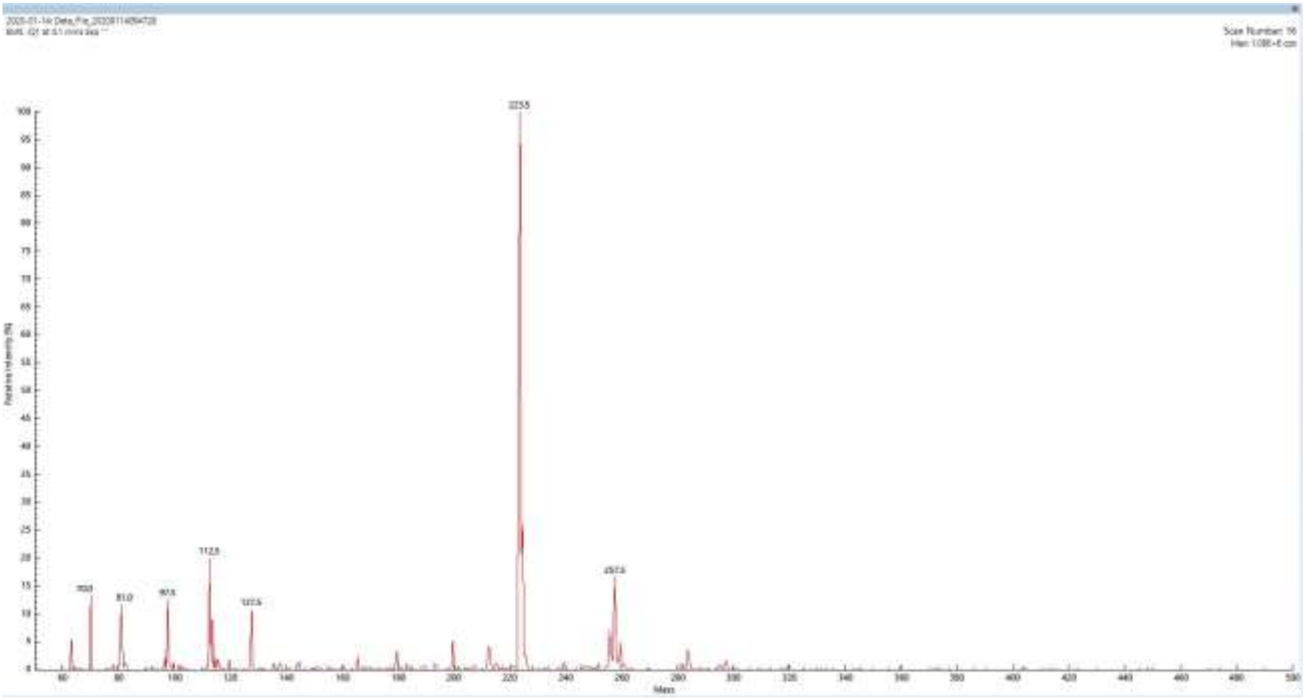

Mass Spectrum 1 ppm Olivetolic Acid - Fragmentation

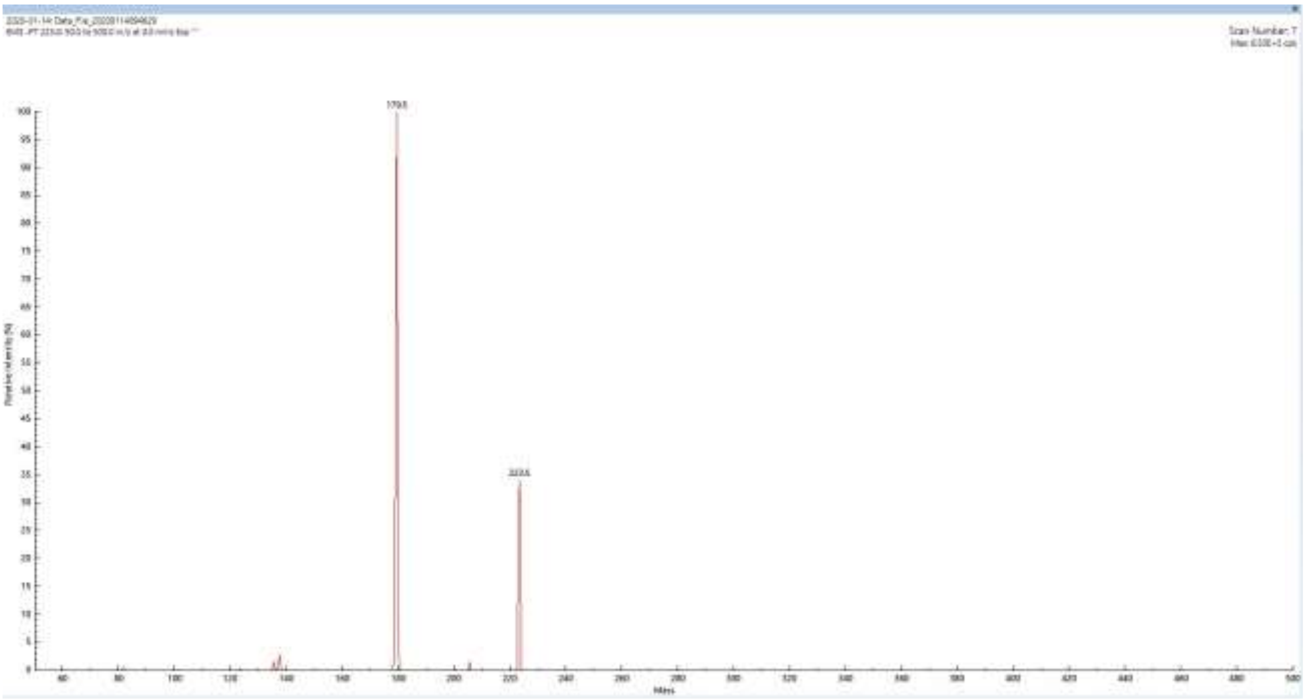

Olivetolic Acid by High Resolution Mass Spectrometry (HRMS)

For: Jingbo Ma (CBEE)
January 15, 2020

Report prepared by: M. LaCourse

Date: January 15, 2020

Report reviewed by: Joshua Wilhide

Date:

Instrumentation & Method

Instrumentation:
- Bruker 12T solariX FT-ICR-MS

MS Method:
- Negative mode ESI ionization
- Fragmentation with collision energy 12V

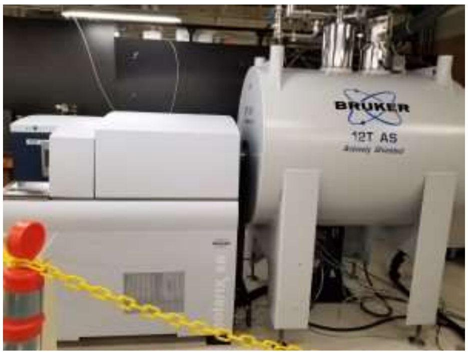

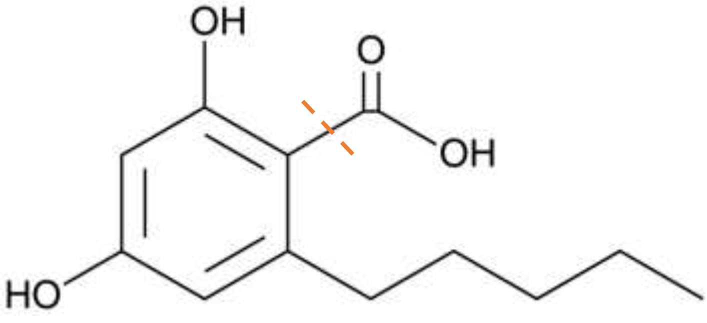

Fragmentation

$C_{12}H_{16}O_4$ – COO → $C_{11}H_{16}O_2$

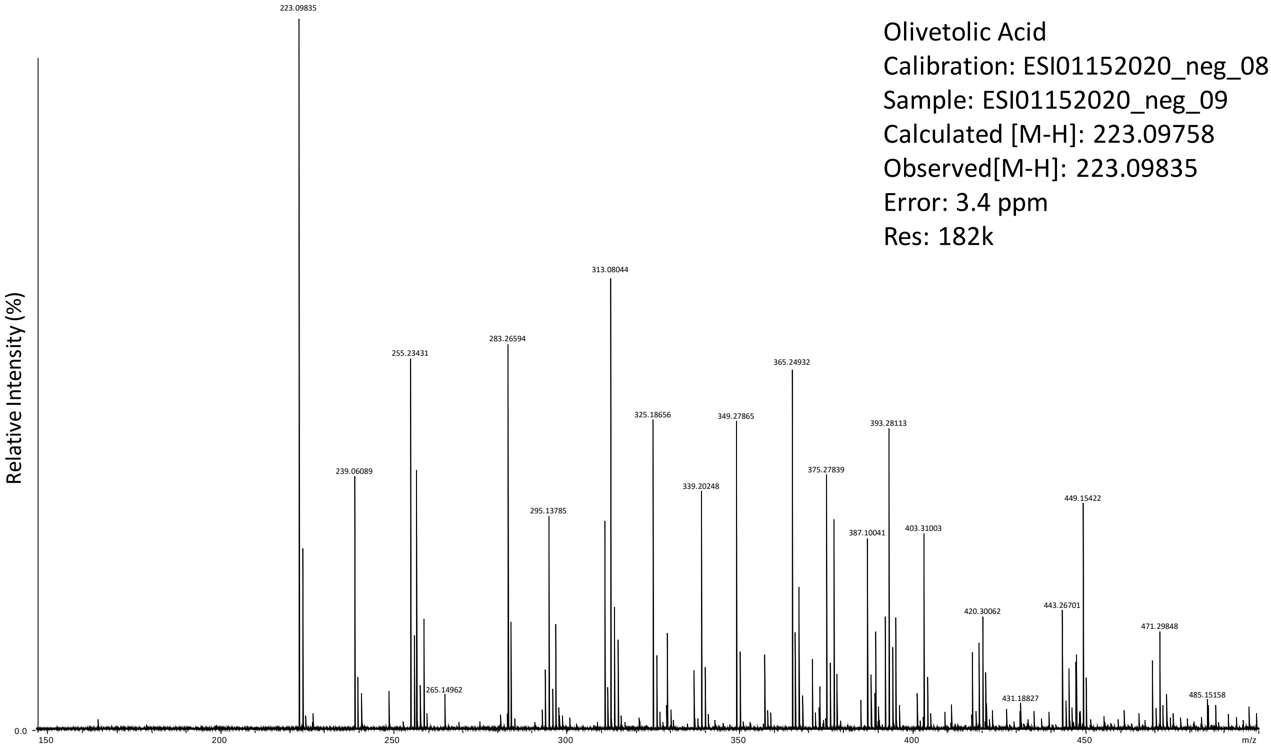

Olivetolic Acid
Calibration: ESI01152020_neg_08
Sample: ESI01152020_neg_09
Calculated [M-H]: 223.09758
Observed[M-H]: 223.09835
Error: 3.4 ppm
Res: 182k

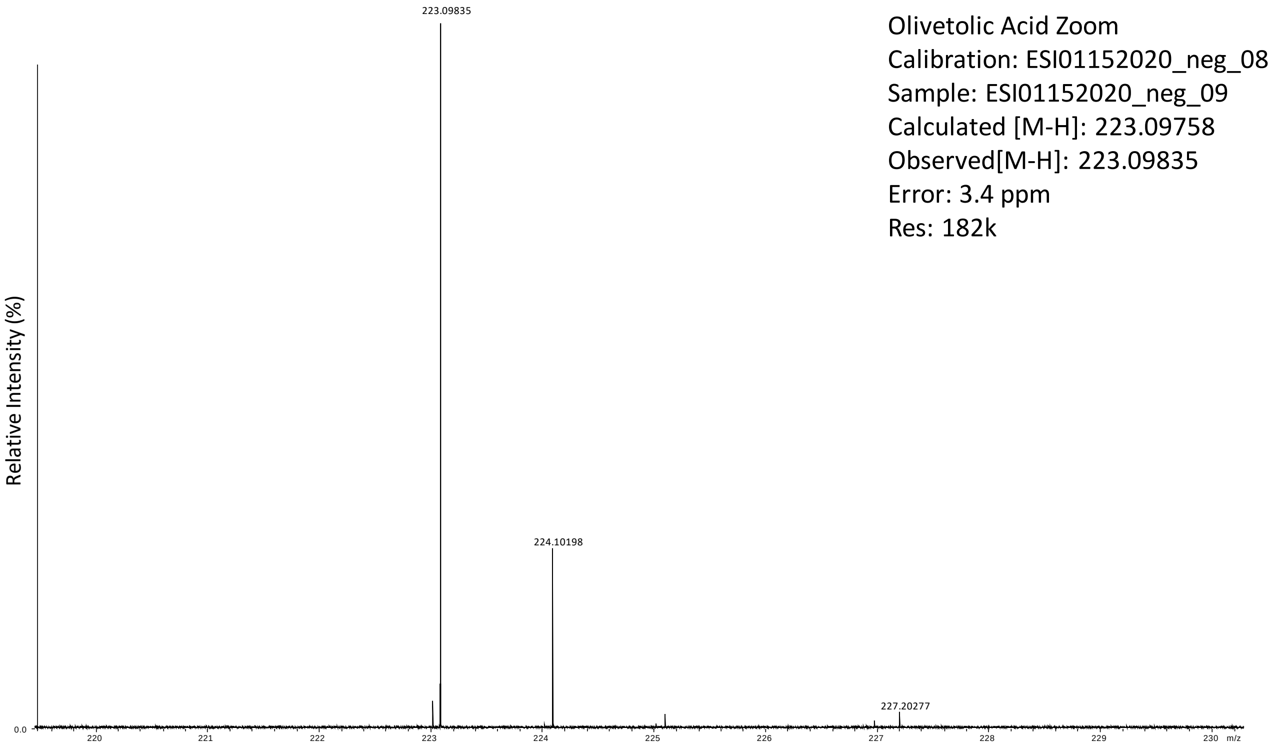
223.09835
224.10198
227.20277
Relative Intensity (%)
Olivetolic Acid Zoom
Calibration: ESI01152020_neg_08
Sample: ESI01152020_neg_09
Calculated [M-H]: 223.09758
Observed[M-H]: 223.09835
Error: 3.4 ppm
Res: 182k

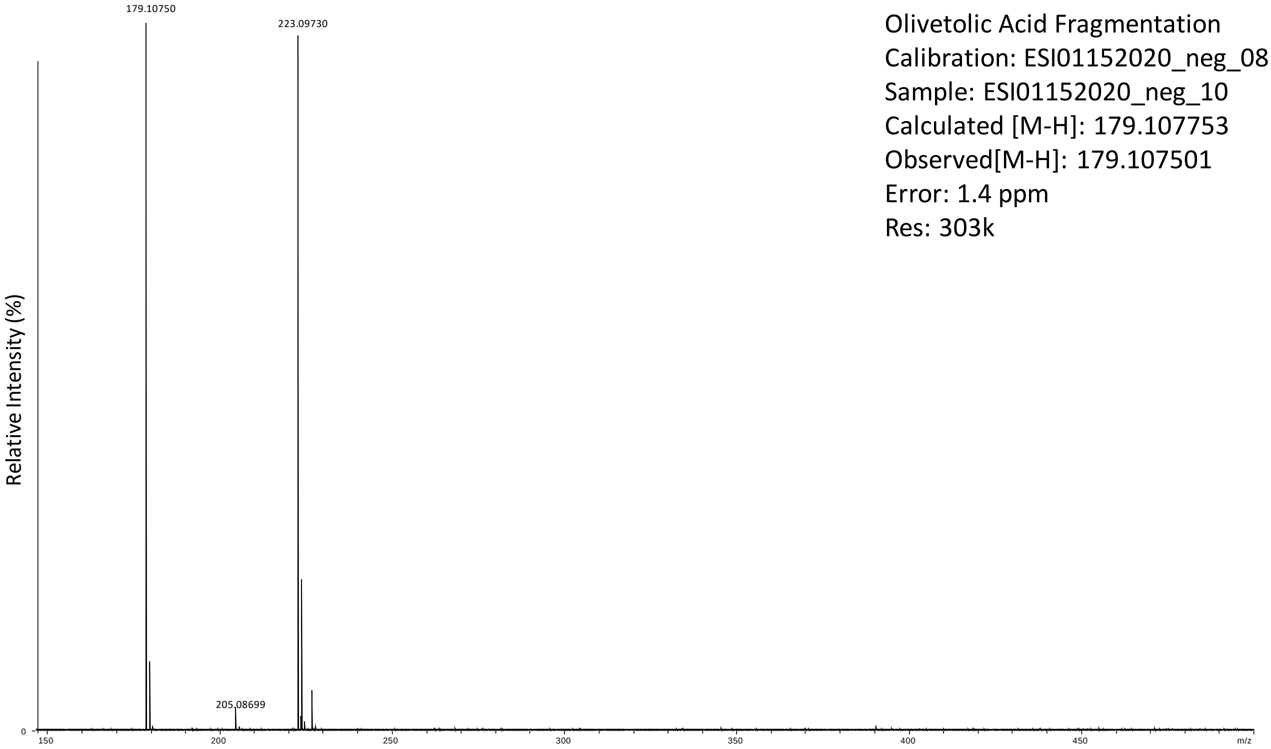
179.10750
223.09730
205.08699
Relative Intensity (%)
Olivetolic Acid Fragmentation
Calibration: ESI01152020_neg_08
Sample: ESI01152020_neg_10
Calculated [M-H]: 179.107753
Observed[M-H]: 179.107501
Error: 1.4 ppm
Res: 303k

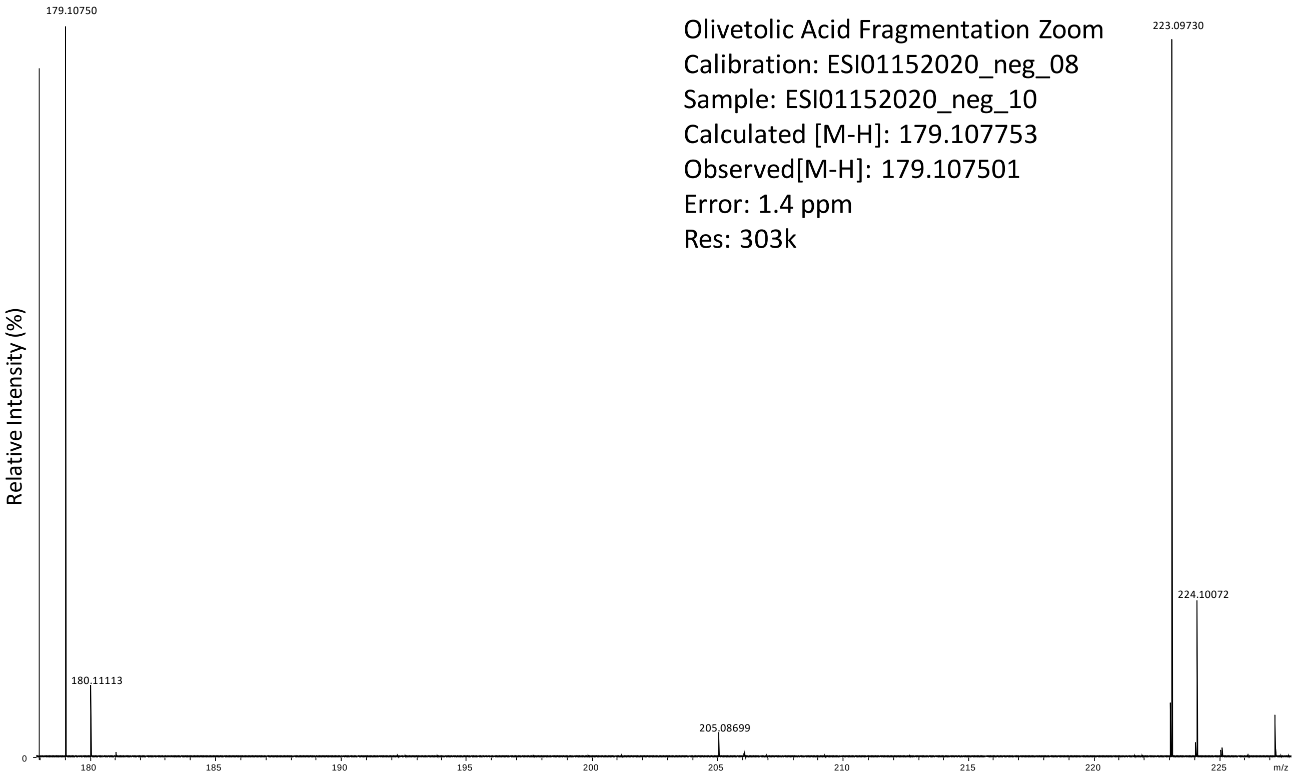

179.10750
180.11113
205.08699
223.09730
224.10072
Olivetolic Acid Fragmentation Zoom
Calibration: ESI01152020_neg_08
Sample: ESI01152020_neg_10
Calculated [M-H]: 179.107753
Observed[M-H]: 179.107501
Error: 1.4 ppm
Res: 303k
Relative Intensity (%)
0
180
185
190
195
200
205
210
215
220
225
m/z
