# Supplementary material for: Biosynthesis of cannabinoid precursor olivetolic acid in genetically engineered Yarrowia lipolytica
Source: Commun Biol. 2022 Nov 12;5:1239. doi: 10.1038/s42003-022-04202-1 (PMC9653464; doi:10.1038/s42003-022-04202-1)
Supplement: Supplementary file 3 — Description of Additional Supplementary Data [file 42003_2022_4202_MOESM3_ESM.docx]

**Description of Additional Supplementary Files**

**File name:** Supplementary Data 1

**Description:** Source data for graphical representations in Figures 2-5.
